# Small bowel Crohn’s disease neglected by gastroenterologists and anorectal surgeons with a 7-year delay in diagnosis: A case report

**DOI:** 10.1097/MD.0000000000039765

**Published:** 2024-09-27

**Authors:** Jiayan Shen, Yingshuang Huang, Weiwei Wang, Rubin Ke, Xiuqin Fan

**Affiliations:** a Department of Gastroenterology, Linping Hospital of Integrated Traditional Chinese and Western Medicine, Hangzhou, Zhejiang, P.R. China; b Department of Pathology, Linping Hospital of Integrated Traditional Chinese and Western Medicine, Hangzhou, Zhejiang, P.R. China.

**Keywords:** body mass index, EEN, intestinal fistula, perianal abscess, SBCD

## Abstract

**Rational:**

Small bowel Crohn’s disease (SBCD) is a common site of Crohn’s disease (CD). However, owing to the anatomical characteristics of the small bowel and the limitations of traditional examination methods, the detection and diagnosis of SBCD remain difficult. Gastroenterologists and anorectal surgeons should pay more attention to improving the early diagnosis rate, so as to improve the prognosis of patients and reduce the probability of surgery due to complications.

**Patient concerns:**

Here, we presented a case of a young male with severe localized pain in the right kidney area and fever but no weight loss or diarrhea, who had a history of perianal abscess surgery 7 years ago and an elevated platelet count reviewing his previous medical examination report.

**Diagnoses:**

SBCD was not diagnosed until complications of intestinal fistula developed 7 years after perianal abscess surgery.

**Interventions:**

Anti-infection treatment was administered due to elevated inflammatory markers and evidence of infection on computed tomography scan, and exclusive enteral nutrition (EEN) was then performed because of the diagnosis of SBCD. Although the infection was absorbed by the treatment with EEN, a laparoscopic modified partial enterectomy was finally performed due to the complication of intestinal fistula.

**Outcomes:**

The patient was discharged on the seventh postoperative day without postoperative complications and started biologic therapy 2 weeks after surgery because he had high-risk factors for postoperative recurrence. The pathological report revealed the involvement of the ileum in CD, and confirmed the existence of the intestinal fistula.

**Lessons:**

Gastroenterologists and anorectal surgeons should be aware that perianal abscess could be the first manifestation of SBCD; even if typical CD manifestations are absent, proper further examinations are necessary based on the comprehensive analysis of clinical data of patients. In addition, the platelet count deserves attention in patients with potentially possible CD. More importantly, it is important to emphasize the importance of EEN in adult CD patients.

## 1. Introduction

Crohn’s disease (CD) is a chronic inflammatory disease that affects all segments of the gastrointestinal tract, more prevalent in developed countries, and has an increasing incidence in China. The prevalence is highest in Europe (322 per 100 000), Canada (319 per 100 000) and the USA (214 per 100 000).^[1]^ The incidence of CD differs by region, ranging from 0 to 29.2 cases per 100,000 patients per year.^[2]^ The most commonly affected intestinal segments are the terminal ileum and colon, 28% frequency in small intestine,^[3]^ with the symptoms evolving in a relapsing and remitting manner. CD can frequently and silently aggravate and cause multiple complications, including fistulas, abscesses, obstruction, and internal bleeding, especially when the involvement is confined to the small intestine. Notably, Small bowel Crohn’s disease (SBCD) is often first diagnosed when these complications occur, and 50% of CD patients undergo an intestinal resection during their lifetime consequently.^[4]^ To date, the difficulty of diagnosis remains the main cause increasing the morbidity and in some cases, even the mortality of these patients. The typical clinical manifestations of CD include frequent abdominal pain, diarrhea, hematochezia, and weight loss, usually occurring between 18 and 35 years.^[2]^ When Crohn’s disease only involves the small intestine, the clinical manifestations are insidious, which leads to difficulties in early diagnosis.

We presented a case of SBCD with a operation history of perianal abscess, which may be the first symptom of CD and also a complication; however, it was not diagnosed as SBCD for the first time until intestinal fistula occurred 7 years later. As a result, gastroenterologists and anorectal surgeons should pay more attention to improving the early diagnosis rate of such patients, so as to improve the prognosis of patients and reduce the probability of surgery due to complications.

## 2. Case presentation

**Figure 1.**
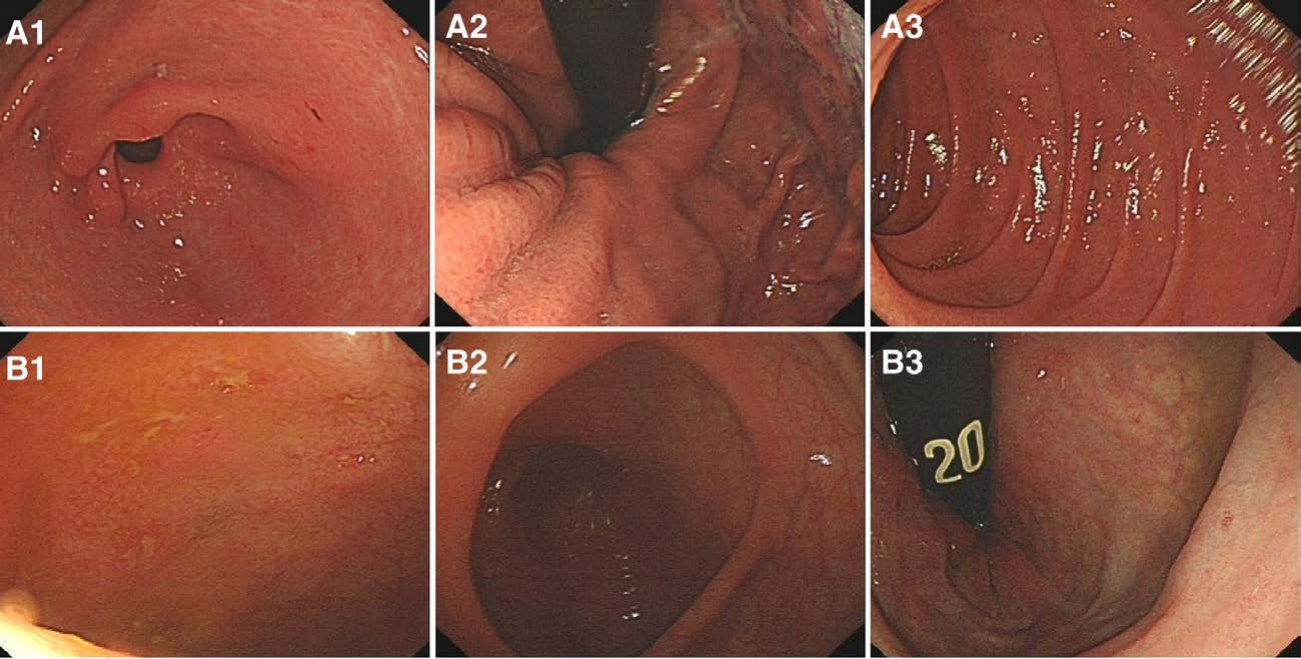
Gastrointestinal endoscopy (A1–3 for gastroscope; B1–3 for colonoscopy). Gastroscopy reveals chronic atrophic gastritis with erosion, and A1–3 represent the gastric antral, fundus, and duodenum, respectively. B1 shows scattered mucosal erosion. B2–3 shows no abnormalities in the cecocolic mucosa.

**Figure 2.**
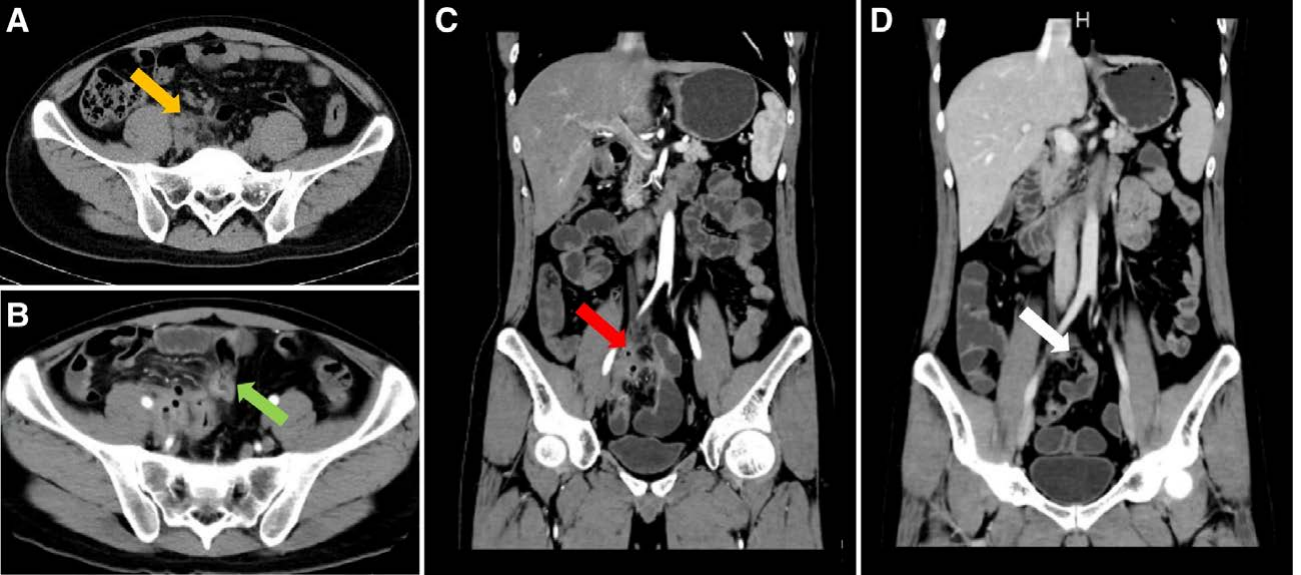
Relevant imaging data during the course of the disease. Abdominal CT scan shows infectious exudation around the mid-ureter (A; orange arrow); contrast-enhanced CT scan shows multiple segmental wall thickening in the small bowel, especially in the ileum, with partial stenosis (B; green arrow). CTE reveals focal small bowel areas of wall thickening and an intestinal fistula (C; red arrow). Reevaluated CTE after 10 weeks of exclusive enteral nutrition reveals absorbed infected lesions and a remaining intestinal fistula (D; white arrow).

**Figure 3.**
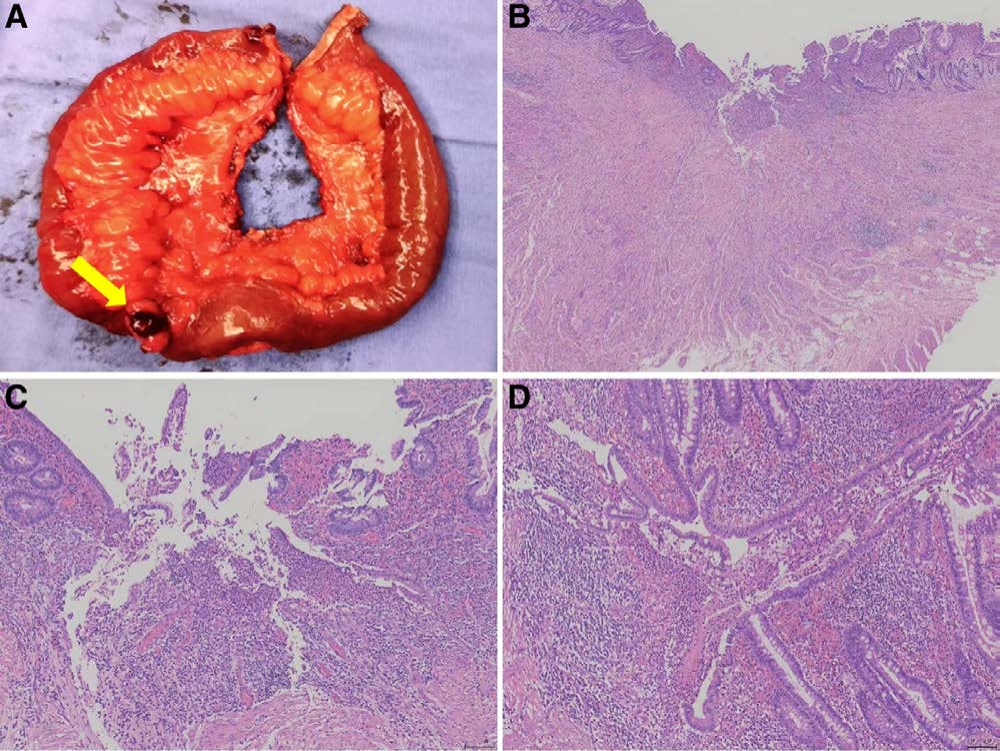
The postoperative pathology. The ileum with fistula (A: yellow arrow), inflammatory cell invasion through all layers of the intestine (B: hematoxylin and eosin stain, object lens × 2), fissured ulcers (C: hematoxylin and eosin stain, object lens × 10) and noncaseating granulomas (D: hematoxylin and eosin stain, object lens × 10).

We present the case of a 30-year-old male with a history of perianal abscess surgery 7 years ago, who presented to the emergency department with severe localized pain in the right kidney area, sudden onset, 7/10 in intensity. He had fever, loss of appetite but no weight loss or diarrhea, with a body mass index of 23.89 kg/m^2^. The patient had abnormal vital signs with a temperature of 38.9 °C and heart rate of approximately 102/min, others were unremarkable. The patient was a nonsmoker. It is worth mentioning that the patient underwent gastrointestinal endoscopy (Fig. 1) approximately one and a half months ago, due to unformed stools 1 to 2 times a day and occasional epigastric pain especially after drinking alcohol since anorectal fistula surgery 7 years ago. Gastroscopy suggested chronic atrophic gastritis with erosion and colonoscopy suggested terminal ileitis without biopsy. More importantly, the patient also underwent colonoscopy due to wound discharge after anal fistula surgery; however, the result only revealed chronic colitis, and there were no special positive pathological results. At that time, he was therefore given mesalazine suppository therapy suggested by an anorectal surgeon for approximately 2 months, without further examination and investigation. Perhaps, this is when the truth passed. Clinical examination revealed positive percussion pain in the right renal area, whereas abdominal pain on deep palpation, rebound tenderness, rigidity, and guarding were absent. Therefore, acute pyelonephritis was suspected. Thus, specific investigations, including complete blood count, erythrocyte sedimentation rate (ESR), serum electrolytes, urinalysis, and computed tomography (CT) were performed, and the results of serum electrolytes returned normal as well as urinalysis results. Other examinations indicated evidence of inflammation; however, the cause was unknown. Specifically, the white blood cell, and ESR were elevated (Table 1, October 14th). Moreover, abdominal CT showed a dilated right ureter and renal pelvis, and the middle segment was not clearly visible with an exudative mass periphery (Fig. 2A). Therefore, he was given intravenous treatment of anti-infection with cefotaxime intravenously for 3 days. Antibiotics were upgraded to cefoperazone sulbactam sodium due to a lack of improvement in blood examination (Table 1, October 17th). In the meantime, a contrast-enhanced CT scan was ordered to confirm the cause of infection, which showed multiple segmental wall thickening in the small bowel, especially in the ileum, partial stenosis, sinus tract formation, and abscess (Fig. 2B). Ob the basis of the contrast-enhanced CT findings, the diagnosis of SBCD was indicated, small intestinal tuberculosis and ulcerative colitis could not be excluded definitely. After reviewing the laboratory and imaging results, a stool sample was sent for calprotectin level testing, which was 983.5 µg/g, while the fecal occult blood test was negative. Further examination including CT enterography (CTE) (Fig. 2C) revealed focal small bowel areas of wall thickening, in keeping with skip lesions found in CD. After 2 weeks of anti-infection treatment, the patient’s blood tests were basically normal, but ESR and fecal calprotectin remained elevated (Table 1, October 30th). The patient then underwent exclusive enteral nutrition (EEN), consisting of 3 weeks of total enteral nutrition by nasal feeding with enteral nutritional suspension, and 7 weeks of oral nutritional supplements with enteral nutritional powder (TP). After 10 weeks of EEN, intestinal CTE, laboratory tests, and imaging tests were reevaluated. ESR decreased to normal, so as fecal calprotectin, and albumin, which is an indicator of nutrient status, increased (Table 1, January 15th). In addition, CTE (Fig. 2D) showed that the infected lesion was almost completely absorbed, but the intestinal fistula remained. Due to the complication of intestinal fistula, laparoscopic modified partial enterectomy was performed. The postoperative period was uncomplicated and the patient was discharged on the 7th postoperative day. The pathological report (Fig. 3) revealed the involvement of the ileum in CD without the development of any neoplasm, and also confirmed the existence of the intestinal fistula. The hematoxylin and eosin staining results were consistent with the pathological manifestations of CD. Considering SBCD as the final diagnosis, the patient started biologic therapy 2 weeks after surgery because of the high-risk factors for postoperative recurrence.

**Table 1:** Laboratory parameters of the patient throughout the course of Crohn’s disease.

Time	WBC	HB	PLT	CRP	ESR	FC	ALB
2023.10.14	18.7	127	307	93.3	63	/	35
2023.10.17	9.6	122	241	118.8	86	983.5	/
2023.10.30	4.73	123	216	1.63	31	62.6	39
2024.01.15	6.3	147	212	1.6	9	＜15	47.6

ALB = albumin, CRP = C-reactive protein, ESR = erythrocyte sedimentation rate, FC = fecal calprotectin, HB = hemoglobin, PLT = platelet, WBC = white blood cell.

## 3. Discussion

To our knowledge, Crohn’s disease is one of the inflammatory bowel disease, which is characterized by transmural pattern inflammation of the intestine and granulomatous features observed on biopsy. This report aims to emphasize the significance of early diagnosis, when an anorectal surgeon encounters a young patient undergoing surgery for a perianal abscess, especially when the postoperative incision does not heal well, whether the patient smokes or not, attention should be paid to the possibility of CD. In addition, past literature has reported increased complication rates after skin tag removal or excisional hemorrhoidectomy in patients who have CD without a known diagnosis of CD (50% vs 9.8%).^[5]^ It is important to stress that reviewing perianal abscess as the first manifestation of CD will be helpful in the diagnosis of such cases in the future, and avoid disease progression even surgery caused by missed diagnosis. Moreover, despite poor compliance with adults and more recommended for children with CD, it is still important to emphasize the importance of EEN in adult patients with CD. As represented in our case, preoperative total EEN was adopted and achieved a good curative effect.

As low-risk countries such as China, Japan, and India have adopted a western lifestyle, the incidence of CD has increased sharply.^[1]^ Up to now, the etiology of the disease remains unclear; it is believed to result from a complex interplay between genetic susceptibility, environmental factors, and the intestinal microflora. Genetic defects, including mutations in NOD2, ATG16L1, LRRK2, XBP1, and IRGM, may be associated with the pathogenesis of CD.^[6]^ In addition, cigarette smoking is associated with a two-times increase in the risk of Crohn’s disease (odds ratio [OR] 1.76; 95% CI 1.40–2.22),^[7]^ and patients diagnosed with Crohn’s disease who continue to smoke were more likely to experience a flare-up (56%) and require a first intestinal resection (54%) compared to nonsmokers^.[8]^ Accordingly, it is reasonable to infer that the presented patient’s lack of smoking habits may have plausibly contributed to the deceleration of disease progression; however, the underlying mechanisms remain unreported.

CD typically presents in early adulthood with frequent abdominal pain, diarrhea, hematochezia, fatigue, and weight loss. However, the presenting symptoms can be heterogeneous and insidious, depending on the severity and location of the inflammation and disease behavior. The patient we reported exhibited atypical symptoms, only presented with shapeless stool 1 to 2 times per day 7 years after perianal abscess surgery. Although gastrointestinal endoscopy yielded limited positive findings indicative of CD, such as the so-called cobblestone pattern, a review of his previous medical examination report revealed an elevated platelet count, suggesting the possibility of inflammation. The involvement of the CD40/CD40L platelet complex in the development of inflammatory bowel disease has been overwhelmingly demonstrated,^[9]^ and the activation of platelet hyperactivity in active CD through the ROS-NLRP3 inflammasome-interleukin-1 β axis was reported recently.^[10]^ However, no physician integrated his past medical history with laboratory tests, and associated with the possibility of CD; therefore, no further examination was conducted for 7 years. Collectively, the platelet count deserves higher attention, but whether it can be used as an independent predictor of CD remains to be investigated.

Because of insidious characteristic of SBCD, especially those involving only the small intestine, just like the case we presented, clinical symptoms occur usually at the time of complications emerged, such as fistulas, abscesses, obstruction, and internal bleeding. Therefore, early diagnosis of SBCD remains difficult, and blood inflammatory indicators such as C-reactive protein and ESR do not parallel well with the fluctuation of the disease, and symptoms do not necessarily correlate with objective assessment of disease activity. Therefore, exploration of biomarkers that can predict disease course is gaining increasing importance.

## 4. Conclusion

Analyzing the journey that was finally diagnosed with SBCD in our patient, it is crucial to note that perianal abscess can be the first manifestation of SBCD. Although endoscopy remains the gold standard for diagnosis, evidence is not always found in colonoscopy biopsies, especially in cases of inflammation located in the small intestine. In addition, the platelet count deserves attention in patients with potentially possible CD, but whether it can be used as an independent predictor of CD remains to be investigated. Early diagnosis and early aggressive treatment result in a better prognosis, and may decrease or avoid surgery in the treatment of SBCD. Thus, research on genes and biomarkers to predict the CD get an urgent priority.

## Acknowledgments

We thank JY Shen’s husband for providing this case and for teaching us a sobering lesson and hope that he will maintain remission in his subsequent disease surveillance. JY Shen would like to thank her colleagues and family members for their support and encouragement.

## Author contributions

**Conceptualization:** Jiayan Shen, Xiuqin Fan.

**Data curation:** Jiayan Shen, Yingshuang Huang.

**Formal analysis:** Jiayan Shen, Yingshuang Huang.

**Investigation:** Jiayan Shen, Weiwei Wang.

**Resources:** Jiayan Shen, Yingshuang Huang, Rubin Ke.

**Supervision:** Xiuqin Fan.

**Writing – original draft:** Jiayan Shen.

**Writing – review & editing:** Jiayan Shen, Yingshuang Huang, Weiwei Wang, Rubin Ke, Xiuqin Fan.
